# Medium-term outcomes after laparoscopic revision of laparoscopic Kasai portoenterostomy in patients with biliary atresia

**DOI:** 10.1186/s13023-021-01835-z

**Published:** 2021-04-30

**Authors:** Yi Ji, Xuepeng Zhang, Siyuan Chen, Yanan Li, Kaiying Yang, Jiangyuan Zhou, Zhicheng Xu

**Affiliations:** 1 Department of Pediatric Surgery, West China Hospital of Sichuan University. #37 Guo-Xue-Xiang, Chengdu, 610041 Sichuan China; 2 Pediatric Intensive Care Unit, Department of Critical Care Medicine, West China Hospital of Sichuan University, Chengdu, 610041 China

**Keywords:** Biliary atresia, Laparoscopic Kasai portoenterostomy, Revision, Outcomes

## Abstract

**Objective:**

To determine whether revision laparoscopic Kasai portoenterostomy (RLKPE) is a viable treatment option for patients with biliary atresia (BA) who had undergone initially successful laparoscopic Kasai portoenterostomy (ILKPE).

**Methods:**

The medical records of 312 patients with nonsyndromic BA who had undergone ILKPE between May 2009 and May 2017 were retrospectively reviewed. The patients were divided into three groups according to their outcomes after ILKPE: group A: 25 patients who had undergone RLKPE; group B: 203 patients who had undergone ILKPE and required no further surgical intervention; group C: 84 patients with failed ILKPE who had either died or required liver transplantation for survival. The 3-year and 5-year survival with native liver (SNL) rates were compared between groups A and B and between groups A and C. Among the 25 patients in group A, the perioperative data of RLKPE were compared with those of ILKPE.

**Results:**

Of the 312 patients who had undergone ILKPE, 228 reached the normal bilirubin concentration range within 6 months postoperatively. Among them, 25 patients with a sudden cessation of bile flow had undergone RLKPE. Adequate biliary drainage, as evidenced by normalized conjugated bilirubin levels, was achieved in 80% of patients who had undergone RLKPE. The perioperative variables, including the operative time, blood loss, rate of conversion to open surgery and complications of RLKPE, were not significantly different between RLKPE and ILKPE. The 3-year and 5-year SNL rates in patients after RLKPE were 64.0% and 52.0%, respectively, which were not significantly different from the corresponding rates of 86.2% and 73.9%, respectively, in patients after unrevised ILKPE (*P* > 0.05).

**Conclusion:**

Our data demonstrated that RPLKE is a viable and effective treatment option in patients with sudden cessation of bile drainage after ILKPE. RPLKE can delay the need for liver transplantation, yielding encouraging medium-term patient outcomes.

## Introduction

Biliary atresia (BA) is a rare disorder, with an incidence of approximately 1 in 10,000 live births worldwide. BA is a progressive fibrous occlusive disease that, if not treated promptly and effectively, may lead to cirrhosis or end-stage liver disease [1]. Although liver transplantation (LT) significantly improves the prognosis of patients with BA, Kasai portoenterostomy remains the standard initial option because of the technical complexity associated with liver transplantation in young children and the shortage of qualified donors [2, 3]. Kasai portoenterostomy can restore excretory and synthetic liver function, enable healthy growth and development, and postpone liver transplantation. In 2002, Esteves [4] developed the laparoscopic Kasai portoenterostomy (LKPE) procedure, which created a new era of minimally invasive surgery for BA. Although a learning curve of LKPE is present, some satisfactory results have been achieved, in which the 3-year and 5-year survival with native liver (SNL) rates after LKPE were not inferior, or even superior, to those after open Kasai portoenterostomy (OKPE) [5–10]. Unfortunately, LKPE is not effective in every patient with BA, as is the case for OKPE. In patients with sudden cessation of bile drainage after initially successful LKPE (ILKPE), the necessity and indications for revision LKPE (RLKPE) have not been previously reported. Herein, we review and analyze our experiences with RLKPE in patients with BA.

## Materials and methods

### Design and study population

This study was approved by the Ethical Committee of the West China Hospital of Sichuan University. The patients’ parents or guardians provided written informed consent. The diagnosis of BA was based on a combination of ultrasonic findings, intraoperative cholangiography findings, and histological examinations of the hepatic parenchyma and extrahepatic bile ducts. Patients with BA splenic malformation, severe cardiac anomalies, or other significant comorbidities that would influence the postoperative course were excluded from the study. The 312 patients with nonsyndromic BA who had undergone LKPE from May 2009 to May 2017 were enrolled in this study. They were divided into 3 cohorts based on their outcome after ILKPE as follows: (1) the 25 patients in group A had undergone RLKPE; (2) the 203 patients in group B had undergone ILKPE and required no further surgical intervention; (3) the 84 patients in group C had failed ILKPE and had either died or required liver transplantation for survival. The indications for RLKPE were the sudden cessation of bile drainage after ILKPE and no improvement after 2 weeks of antibiotic treatment. Patients were not recommended for RLKPE if the bile drainage failure lasted more than 60 days or if they had severe ascites or aggravated liver function. The 3-year and 5-year SNL rates were compared between groups A and B and between groups A and C. Among the patients in group A, the perioperative data, rates of clearance of jaundice (CJ) and cholangitis of RLKPE were compared with those of ILKPE.

### Operative technique

#### LKPE procedure

The LKPE procedure was similar to that described in a previously published paper [5–7]. Briefly, a percutaneous suture was used to snare the round and falciform ligament and retract the liver. The portal plate over the bifurcation of the main portal vein could be found along the common hepatic duct. The resection level of the fibrous cone depended on the presence of abundant bile-like juice over the fibrous stump. Bleeding from the fibrous remains of the portal plate was controlled by direct compression with gauze pads. The Roux loop was delivered to the hilum through a retrocolic path. One layer of end-to-side portoenterostomy (diameter of the anastomosis: 1.0–1.5 cm) was performed with interrupted 5–0 absorbable sutures. A drain was inserted into the foramen of Winslow.

#### RLKPE procedure

The trocar placement and pneumoperitoneum pressure settings were the same as those in LKPE, as mentioned above. The other steps of RLPKE were as follows: (1) removing the greater omentum adhered to the porta hepatis; (2) partly dismantling the anastomosed Roux limb adjacent to the porta hepatis; (3) resecting the granulation tissue; (4) dissecting the hilar fibrous stump layer by layer until abundant bile-like juice was observed; (5) performing end-to-end portoenterostomy after trimming the tail of the Roux limb.

### Management algorithm after surgery

Postoperatively, the BA patients in the 3 groups received the same medical treatment protocol. Antibiotic therapy was continued intravenously for 25–30 days in the hospital. Methylprednisolone was administered intravenously 5 days postoperatively at a dose of 5 mg/kg/day initially and was reduced by 1 mg/kg/day every 3 days for 2 weeks or longer until a normal total bilirubin value was reached. Sulfamethoxazole and cephalosporin antibiotics were orally administered alternately weekly until 1 year of age. Ursodeoxycholic acid and hepatoprotective tablets were used until 3 years of age.

### Definitions

The operative time (ORT) was calculated as the length of time between the skin incision and closure. Any perioperative complications (APOC) was defined as a complication that occurred during the perioperative period, including wound infection, umbilical hernia, intestinal anastomotic fistula, and adhesive intestinal obstruction. CJ was defined as a total bilirubin level < 1.2 mg/dL within 6 months postoperatively. Cholangitis was defined as an elevated serum bilirubin (> 2.5 mg/dL), leukocytosis with a left shift, and normal to acholic stools in a febrile patient (> 38.0 °C). The postoperative outcomes were followed through outpatient clinic evaluations and were excluded if the patients had undergone liver transplantation or had died.

### Statistics

The software applied for statistical calculations was SPSS 22.0 for Windows 10.0 (SPSS Inc., Chicago, IL, USA). The demographic data of BA patients were compared using one-way analysis of variance and chi-squared test. The difference in the SNL rate among the patients was analyzed by the Kaplan–Meier method with endpoints of death or liver transplantation and compared using the log-rank test. A *P* value < 0.05 was considered statistically significant.

## Results

### Demographic and clinical characteristics of all the patients in the three groups

From May 2009 to May 2019, 312 patients with nonsyndromic BA had undergone ILKPE. The demographic and clinical characteristics of all the patients in the three groups are presented in Table 1. The median age at operation was 82.8 ± 19.4 days, with a male proportion of 32.1%. The proportions of types I, II, and III and cystic BA were 1.6%, 4.8%, 82.4% and 11.2%, respectively. The median follow-up time was 67.2 ± 25.5 months. The rates of CJ and cholangitis after ILKPE were 73.1% and 58.9%, respectively. The 3-year and 5-year SNL rates were 62.6% and 54.1%, respectively.

**Table 1:** Demographic and clinical characteristics of all patients who underwent LKPE^*^

Characteristics	Group A^†^ N = 25	Group B^†^ N = 203	Group C^†^ N = 84	Total N = 312	*P*^‡^
Sex, n (%) Male	9 (36%)	65 (32%)	26 (31.0%)	100 (32.1%)	0.69
Age at LKPE (days)	76.7 ± 15.9	81.8 ± 18.7	87.1 ± 21.5	82.8 ± 19.4	0.53
Classification of BA, n (%)					
I	1 (4%)	3 (1.5%)	1 (1.2%)	5 (1.6%)	0.37
II	1 (4%)	12 (5.9%)	2 (2.4%)	15 (4.8%)	0.70
III	22 (88%)	156 (76.8%)	79 (94.0%)	257 (82.4%)	0.20
Cystic	1 (4%)	32 (15.8%)	2 (2.4%)	35 (11.2%)	0.12
Follow-up duration (months)	63.9 ± 26.0	67.9 ± 25.6	66.4 ± 25.2	67.2 ± 25.5	0.81

^*^Values are presented as n (%) or median. LKPE: laparoscopic Kasai portoenterostomy; CJ: clearance of jaundice

^†^Group A: patients who underwent revision LKPE; group B: patients who underwent initially successful LKPE; group C: patients who experienced failed LKPE

^‡^*P*: comparison between group A and group B

### Comparison between groups A and B

**Fig. 1:**
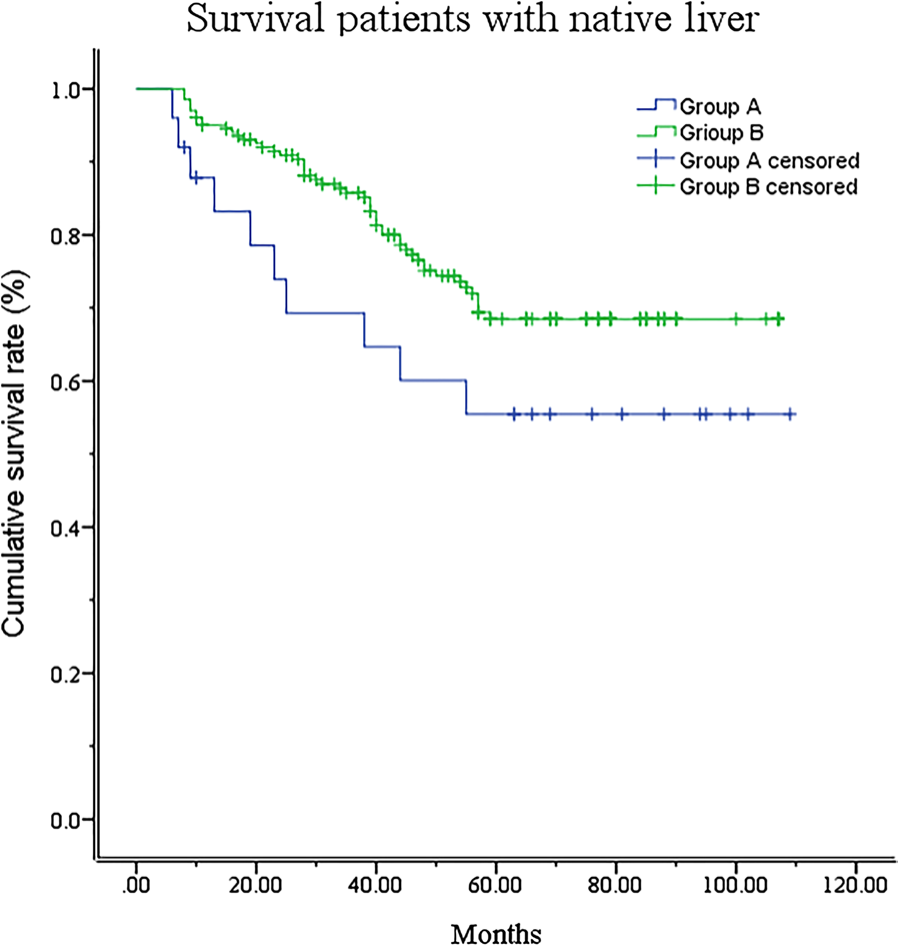
Kaplan–Meier analysis showed that the survival of patients with native livers was not significantly different between groups A and B (*P* > 0.01). Group A: patients who had undergone revision LKPE; group B: patients who had undergone initially successful LKPE

In group A, 25 patients had undergone RLKPE. The male proportion, age and follow-up time were not significantly different between groups A and B. The 3-year and 5-year SNL rates were 64.0% and 52.0%, respectively, in group A and were not significantly different from 86.2% and 73.9%, respectively, in group B (*P* > 0.05) (Fig. 1).

### Comparison of the perioperative variables and rates of CJ and cholangitis between ILKPE and RLKPE in 25 patients

The demographic and clinical characteristics of the 25 patients who had undergone both ILKPE and RLKPE are presented in Table 2. The ORT, blood loss, conversion rate and incidence of AOPC during ILKPE were not significantly different from those during RLKPE. The rates of CJ and cholangitis after ILKPE were 100% and 64.0%, respectively, and were significantly different from the 80.0% and 28.0%, respectively, after RLKPE (*P* < 0.01).

**Table 2:** Demographic and clinical characteristics of the 25 patients who underwent both ILKPE and RLKPE

Characteristic	ILKPE	RLKPE	*P*
Age at LKPE, (days)	76.7 ± 15.9	159.8 ± 50.4	0.00
ORT, (min)	241.1 ± 32.4	179.6 ± 29.5	0.75
Conversion rate, n (%)	0 (0.0)	1 (4.0)	0.31
Median blood loss, (ml)	30.4 ± 19.2	23.0 ± 11.0	0.07
APOC, n (%)	2 (8.0)	1 (4.0)	0.55
CJ, n (%)	25 (100.0)	20 (80.0)	0.00
Cholangitis, n (%)	16 (64.0)	7 (28.0)	0.00

Values are presented as n (%) or median. LKPE: laparoscopic Kasai portoenterostomy. ILKPE: initially successful laparoscopic Kasai portoenterostomy. CJ: clearance of jaundice. ORT: operative time. APOC: any perioperative complication

## Discussion

Experiences with revision for BA patients with recurrent jaundice after initial OKPE or ILKPE have rarely been reported. In two studies, the CJ rates of 60% [11] and 83.3% [12] were obtained after revision of OKPE for patients with initially successful OKPE. Naruhiko et al. showed good results of RLKPE for patients with recurrent jaundice after initial OKPE. The authors found that 10/12 patients had normal bilirubin concentrations after RLKPE [13]. In our study, 20/25 (80%) patients with sudden cessation of bile drainage achieved normal bilirubin concentrations after RLKPE. Although the 3-year and 5-year SNL rates in patients who had undergone RLKPE were lower than those found in patients after unrevised ILKPE, they are sufficiently high to deserve consideration. These satisfactory results were attributed to a rigorous selection of candidates with poor jaundice reduction after the initial Kasai operation. If the indications for revision were relaxed, the postoperative results would be different. However, we cannot exclude the possibility that the absence of a statistical significance between RLKPE and ILKPE regarding SNL rates is because of the small sample of RLKPE. In a survey from the Japanese Biliary Atresia Registry, the revision rate was 21% and the CJ rate was 35% among 2630 BA patients after the failed initial OKPE from 1989 to 2011 [10].

The indications for RLKPE at our center were bile drainage that stopped abruptly after ILKPE and no improvement after 2 weeks of antibiotic treatment. However, a consensus has never been reached concerning the optimal timing of revision worldwide. Some reports have shown that revision could be effective for patients in whom jaundice suddenly recurred after a favorable initial reduction, irrespective of the time since the initial surgery [14, 15]. However, Shirota suggested that the revision should be performed as soon as irreversible jaundice is recognized [12]. In our study, the time interval from the cessation of bile drainage to revision was 21.9 ± 8.0 days, which should be shortened to avoid further liver damage. RLKPE is not recommended for patients with bile drainage failure for more than 60 days or patients with severe ascites or aggravated liver function. For patients older than 1 year with sudden bile drainage failure, antibiotics should be administered first, and liver transplantation is recommended if conservative treatment is ineffective.

Technically, RLKPE is relatively easy to perform, partly due to the omission of the Roux limb anastomosis, which is a time-consuming step in ILKPE. Under the magnified and clear view of the laparoscope, the Roux limb is easily identified by only removing the greater omentum adhered to the porta hepatis [15–18].

However, some authors were reluctant to repeat the Kasai operation because of the possibility of a high incidence of perioperative complications (e.g., uncontrolled intraoperative bleeding) and a longer ORT [13]. Perineal adhesion was more common in patients who had undergone RLKPE. Specifically, in the vicinity of the porta hepatis, dense fibrous granulation tissue sometimes may result in unanticipated bleeding and damage to the Roux limb [11]. In some cases, oozing bleeding from the remains of the hilar fibrous plate is difficult to stop under laparoscopy. Additionally, accidental portal hemorrhage from iatrogenic injuries may place patients at a great risk. In our study, however, fewer peritoneal adhesions around the porta hepatis were observed during RLKPE, at least partly due to the laparoscopic technique used for ILKPE. Under laparoscopy, the image could be zoomed in, and the portal vein could be easily recognized, likely helping to protect it from accidental injuries when dissecting the hilar fibrous plate [19]. Active bleeding at the fibrous stump can be stopped by compression with a gauze pad for a period. In the event of uncontrolled hemorrhage, conversion to OKPE should be adopted without taking risks to ensure the safety of the patients. The above measures adopted in our hospital may make RLKPE safer. In our study, the blood loss volume, conversion rate, ORT and incidence of APOC of RLKPE were not significantly different from those of ILKPE. The technique of RLKPE may be used repeatedly for revision in patients with recurrent jaundice postoperatively.

Three limitations of our study deserve comment. First, the evaluation of RLKPE was based on retrospective data. Second, only a few cases of RLKPE were available compared with the number of unrevised ILKPE cases. Nonetheless, we recommend RLKPE as the preferred surgical treatment for patients with recurrent jaundice after the initial Kasai operation. If the time interval between the cessation of bile drainage and revision is shortened, the postoperative outcomes could be improved. Third, the lack of histological examination of both primary and redo fibrous cone at porta hepatis is another limitation of this study.

## Conclusions

RLKPE is a feasible, safe and effective procedure to treat patients with recurrent jaundice after ILKPE. However, studies with a larger number of patients and a longer follow-up time are needed to confirm the advantages of this technique.

## Data Availability

The datasets used and/or analyzed during the current study are available from the corresponding author on reasonable request.

## Abbreviations

BA: Biliary atresia
LKPE: Revision of laparoscopic Kasai portoenterostomy
ILKPE: Initially successful laparoscopic Kasai portoenterostomy
SNL: Survival with native liver
LT: Liver transplantation
OKPE: Open Kasai portoenterostomy
CJ: Clearance of jaundice
ORT: Operative time
APOC: Any perioperative complications
